# Psychiatric symptoms, risk, and protective factors among university students in quarantine during the COVID-19 pandemic in China

**DOI:** 10.1186/s12992-021-00663-x

**Published:** 2021-01-25

**Authors:** Shufang Sun, Simon B. Goldberg, Danhua Lin, Shan Qiao, Don Operario

**Affiliations:** 1 Department of Behavioral and Social Sciences, Brown University School of Public Health, Providence, RI USA; 2 Mindfulness Center at Brown University, Providence, RI USA; 3 Department of Counseling Psychology, University of Wisconsin-Madison College of Education, Madison, WI USA; 4 Institute of Developmental Psychology, Beijing Normal University, Beijing, China; 5 Department of Health Promotion, Education, and Behavior, University of South Carolina Arnold School of Public Health, Columbia, SC USA

**Keywords:** COVID-19, Psychiatric symptoms, Stigma, Young adult, China

## Abstract

**Background:**

The COVID-19 pandemic has made unprecedented impact on the psychological health of university students, a population vulnerable to distress and mental health disorders. This study investigated psychiatric symptoms (anxiety, depression, and traumatic stress) during state-enforced quarantine among university students in China (*N* = 1912) through a cross-sectional survey during March and April 2020.

**Results:**

Psychiatric symptoms were alarmingly prevalent: 67.05% reported traumatic stress, 46.55% had depressive symptoms, and 34.73% reported anxiety symptoms. Further, 19.56% endorsed suicidal ideation. We explored risk and protective factors of psychological health, including demographic variables, two known protective factors for mental health (mindfulness, perceived social support), four COVID-specific factors (COVID-19 related efficacy, perceived COVID-19 threat, perceived COVID-19 societal stigma, COVID-19 prosocial behavior) and screen media usage. Across symptom domains, mindfulness was associated with lower symptom severity, while COVID-19 related financial stress, perceived COVID-19 societal stigma, and perceived COVID-19 threat were associated with higher symptom severity. COVID-19 threat and COVID-19 stigma showed main and interactive effects in predicting all mental health outcomes, with their combination associated with highest symptom severity. Screen media device usage was positively associated with depression. Female gender and COVID-19 prosocial behavior were associated with higher anxiety, while COVID-19 self-efficacy associated with lower anxiety symptoms.

**Conclusions:**

Findings suggest high need for psychological health promotion among university students during the COVID-19 pandemic and inform an ecological perspective on the detrimental role of stigma during an emerging infectious disease outbreak. Interventions targeting multi-level factors, such as promoting mindfulness and social support at individual and interpersonal levels while reducing public stigma about COVID-19, may be particularly promising. Attending to the needs of disadvantaged groups including those financially impacted by COVID-19 is needed.

## Introduction

The 2019 novel coronavirus virus (COVID-19) pandemic has become a major public health crisis globally. In addition to the physical health consequences, public health authorities across the globe have expressed growing concerns over an international mental health crisis due to quarantine, social isolation, financial strain, and the threat of infection [49, 62]. The mental health impact of a pandemic of this scale is yet to be understood, and such an understanding is valuable for characterizing and ultimately addressing the psychological fallout of the current and future pandemics in the age of increased global and local human mobility as well as increasing a basic scientific understanding of the psychiatric consequences of extreme stress [29, 56].

Across the globe, university students are not considered a priority group in terms of COVID-19 prevention, yet they may be a particularly vulnerable population to the psychological consequences of the COVID-19 pandemic. Mental health of young people has long been recognized as a global public health challenge [47]. Developmentally, many mental disorders have symptoms that first occur during young adulthood, which can negatively impact developmental trajectories through reduced educational achievement, increased substance use, and poor health behaviors [5, 47]. In China, lockdowns through “Level-1 Public Health Emergency Response” across the nation occurred between January 23rd, 2020 and January 29th, 2020, a time when students were spending the traditional Chinese New Year holiday (i.e., Spring Festival) with their families during the winter break [41]. The pandemic has led to massive disruption in the lives and education of university students in China, through prolonged school closure, and transition to internet-based learning, and social isolation from peers during state-enforced quarantine [43]. Available evidence has indeed noted elevated psychiatric symptoms among university students in China during quarantine. Two studies conducted in two different universities found rates of clinically elevated anxiety symptoms to be 15.4% [59] and 24.9% [14] during the early outbreak of COVID-19. Another study with students from six universities in southwest China during February 2020 found 2.7 and 9.0% students reported moderate to severe forms of traumatic stress and depression, respectively [57]. While highlighting high levels of distress, more research is needed to fully understand the psychological impact of the COVID-19 pandemic among university students in China. Beyond estimating prevalence, research identifying risk and protective factors is essential for increasing scientific understanding of the varied psychological reactions to large-scale infectious disease outbreaks, provide guidance to policies and intervention strategies, and ultimately move the bell-shaped curve of population mental health among students who may suffer from the psychological consequences of massive quarantine measures in China and other low- and middle-income country (LMIC) context.

The current study investigates the prevalence of mental health issues among university students in China during state-ordered quarantine and explores risk and protective factors. Three domains of psychiatric symptoms were surveyed, including anxiety, depression, and traumatic stress. Several demographic and contextual factors were explored as symptom correlates, including socioeconomic status, as COVID-19 has disproportionately impacted families from low-income backgrounds and financial hardship may affect mental health [1]. Two widely studied protective factors were explored, including mindfulness and perceived social support. Mindfulness, defined as “paying attention in a particular way, on purpose, in the present moment, and nonjudgmentally” ([31], p. 4), has been consistently identified as a protective factor for psychological distress [11, 25, 32]. Perceived social support is another established protective factor for well-being [8, 28], and it may be particularly crucial during the COVID-19 pandemic due to increased social isolation. Screen media usage was explored as a potentially relevant factor for the mental health of young adults in the pandemic context. Scholars have raised concerns regarding increased internet and smartphone addiction during the COVID-19 pandemic and the negative mental health impact of increased usage [24, 56]. However, to our knowledge, no empirical research has explored its role.

In addition, four COVID-19 related factors were explored as potential predictors, including COVID-19 prosocial behavior, COVID-19 self-efficacy, perceived COVID-19 threat, and perceived COVID-19 societal stigma. Altruistic and prosocial social behavior appears to promote well-being for the helpers [18, 39]. From an evolutionary perspective, prosocial behavior in response to public health threats can be advantageous for both the group and individual [34]. COVID-19 self-efficacy was explored as a predictor of mental health, as belief in one’s capacity to prevent COVID-19 and take necessary steps for treatment may facilitate an increased sense of control in an evolving outbreak [48]. Consistent with prior work showing perceived SARS threat predicted emotional exhaustion among frontline nurses during the SARS outbreak [23], we examined perceived COVID-19 threat (i.e., perception of one’s likelihood of contracting COVID-19) as a risk factor. Stigma was also examined as a potential factor related to mental health problems, as the early phase of an emerging outbreak tends to be characterized by intense disease-related stigma and fear due to its evolving nature and scientific uncertainties [20, 48]. A recent study found 90% of respondents in China exhibited discriminatory attitudes toward people and regions associated with the COVID-19 outbreak [27]. However, the role of societal stigma of an emerging infectious disease (i.e., perceived negative public attitude toward people and regions associated with the outbreak) on mental health outcomes of a general population is still unknown. Lastly, we examined potential interaction between perceived COVID-19 threat and perceived COVID-19 societal stigma. This was informed by the ecological model (person X context) [9, 17]. Given the large-scale impact of COVID-19 reaching all individuals in the society, perceived COVID-19 societal stigma (a environmental-level factor) may amplify the association between perceived personal threat of COVID-19 and mental health outcomes.

We hypothesized that the following would function as risk factors, indicated by their positive association with psychiatric symptoms: COVID-related financial stress, screen media usage, perceived COVID-19 threat, perceived COVID-19 societal stigma. We also hypothesized an interactive relationship between perceived threat and perceived stigma, such that the link between perceived threat and psychiatric symptoms is increased in the presence of perceived stigma. We hypothesized the following as protective factors, indicated by their negative association with psychiatric symptoms: mindfulness, perceived social support, and COVID-19 prosocial behaviors.

## Methods

### Study design and sampling

Recruitment took place online through advertisements on websites and WeChat-based platforms targeting Chinese university students. This included advertisements distributed by academic advisors in WeChat-based group forums for students in 19 Chinese universities located in various regions of China (seven in south central China, six in north China, five in east China, and one in south west China); all of these universities have students from various provinces. Advertisements encouraged students to distribute the study information to their peers in other universities. Data collection occurred between March 20th and April 10th 2020, approximately 2 months following the official announcement of the COVID-19 outbreak in China (January 20th, 2020) while people were under state-enforced strict quarantine. The study employed a cross-sectional research design.

### Inclusion criteria

Inclusion criteria for participation included (a) being at least 18 years of age, (b) currently enrolled in a Chinese college or university as an undergraduate or graduate student, and (c) fluency in the Chinese language.

### Ethical consideration and approval

Eligibility criteria and a consent form were provided on the survey’s welcome page. Participants were encouraged to take the survey on a personal device (e.g., computer, phone) in a private space. Participants received no monetary compensation. Following completion of the survey, participants were provided with suggestions for coping with psychological distress during the COVID-19 pandemic. The study was approved by the Institutional Review Board at Beijing Normal University. Data were collected via an anonymous online survey.

### Instruments

The survey included demographic information (e.g., age, gender, region, socioeconomic status). Participants were asked how much financial stress the COVID-19 pandemic has brought to their family, from 1 (*no financial stress*) to 5 (*significant financial stress*). A single-item question assessed screen media usage: in the past 2 weeks, outside of school and work time, *how many hours daily* on average have you spent on screen media (e.g., phone, computer)?

Anxiety symptoms were measured by the 7-item Generalized Anxiety Disorder Scale (GAD-7; Chinese version) [54, 65], a widely used screening tool for common anxiety disorders (e.g., Generalized Anxiety Disorder, Panic Disorder). GAD-7 assesses symptoms of anxiety in the past 2 weeks (e.g., “feeling nervous, anxious, or on edge,” “feeling afraid as if something awful might happen”), from 0 (*not at all*) to 3 (*nearly every day*). Recommended clinical cut-off values are 5–9 (mild), 10–14 (moderate), and ≥ 15 (severe) [54]. The scale has demonstrated good reliability and validity in outpatient settings in China [65]. Cronbach’s *α* was 0.96.

Depression was measured by the Patient Health Questionnaire-9 (PHQ-9; Chinese version), a depression screening tool [33, 61] that assesses depressive symptoms in the past 2 weeks. Each item reflects one of the nine DSM-IV criteria for major depressive episode (e.g., “little interest or pleasure in doing things”) [4], from 0 (*not at all*) to 3 (*nearly every day*). Recommended clinical cut-off values are 5–9 (mild), 10–14 (moderate), 15–19 (moderately severe), and ≥ 20 (severe) [33]. The scale has demonstrated good reliability and validity among the general population in China [61]. Cronbach’s *α* was 0.93.

COVID-19 related traumatic stress was assessed by the Impact of Events scale (IES; Chinese version) [30, 66], a 15-item, widely used measure of event-specific distress. Participants were asked to indicate symptom severity in the context of the COVID-19 pandemic, from 0 (*not at all*) to 5 (*often*). Items assessed intrusion and avoidance clusters of posttraumatic stress disorder (PTSD) (e.g., “pictures about it popped into my mind,” “I tried not to talk about it.”). Recommended clinical cut-off values are 9–25 (mild), 26–43 (moderate), and ≥ 44 (severe). The Chinese version has been used following natural disasters (e.g., earthquake) and has demonstrated good reliability and validity [60, 66]. Cronbach’s *α* was 0.95.

Mindfulness was measured by the Chinese version of the Mindful Attention Awareness Scale (MAAS) [11, 19]. The scale consists of 15 items and assesses dispositional mindfulness, namely receptive awareness of and attention to experience in the present moment (e.g., “I find it difficult to stay focused on what’s happening in the present.”). Participants indicated frequency from 1 (*almost always*) to 6 (*almost never*), with a higher score indicating higher dispositional mindfulness. The MAAS has demonstrated good reliability and validity among Chinese people [19]. Cronbach’s *α* was 0.94.

Perceived social support was measured by an adapted version of the Multidimensional Scale of Perceived Social Support (MSPSS)- Chinese version [67]. The original MSPSS assesses perceived social support from three resources (family, friends, significant other). Considering that not all young adults are in a romantic relationship, only the eight items assessing perceived social support from family and friends were used (e.g., “my family really tries to help me,” “I can count on my friends when things go wrong”), which participants rated on a 5-point Likert scale (*1 = strongly disagree; 5 = strongly agree*). Cronbach’s *α* was 0.93.

COVID-19 prosocial behavior was assessed by items adapted from the 4-item Empathic Responding to SARS scale [35] and Prosocialness Scale [15]. Specifically, all four items on the Empathic Responding to SARS scale were adapted to the COVID-19 context (e.g., “Tried to understand other people’ concerns about SARS” was changed to “Tried to understand other people’s concerns about COVID-19”). Five out of the sixteen items from the Prosocialness Scale that involved specific behaviors were selected for adaptation to the COVID-19 context (e.g., “I try to console those who are sad” was changed to “Tried to console those who were sad due to COVID-19”). Participants rated a total of nine items assessing prosocial behavior specific to COVID-19, ranging from 1 (*strongly agree*) to 5 (*strongly disagree*). This newly adapted scale has not been validated. Exploratory factor analysis (EFA) and confirmatory factor analysis (CFA) indicated a one-factor structure, with all items having sufficient loadings (≥ .40, all items had a standardized loading > .70). Cronbach’s *α* was 0.93.

COVID-19 Self-Efficacy was measured through an adapted version of the Ebola-related Self-Efficacy scale [13]. Five items assessed perceived ability in adhering to COVID-19 prevention measures (e.g., “I am confident that I can understand health instructions about COVID-19 prevention”) rated as 1 (*strongly disagree*) to 4 (*strongly agree*). Cronbach’s *α* was 0.90.

Perceived COVID-19 societal stigma was measured via an adapted version of the Perceived External Stigma Subscale of the Ebola-related Stigma Questionnaire [46], which itself was derived from Berger’s HIV Stigma Scale [6]. Six items assessed perceived societal stigma against COVID-19, rated as 1 (*strongly disagree*) to 5 (*strongly agree*). Sample items include “most people are afraid of a person who has had COVID-19 or from regions severely affected by COVID-19”, “most people think that a person who has had COVID-19 is disgusting.” Higher total scores indicate higher levels of stigma. Cronbach’s *α* was 0.91.

Perceived COVID-19 threat was adapted from a measure assessed Chinese people’s perceived threat by SARS [35]. Seven items assessed perceived infection threat by COVID-19 (e.g., “I don’t think I could get COVID-19”, “I think COVID-19 will threaten my health”) rated as 1 (*strongly disagree*) to 4 (*strongly agree*). Higher total scores indicate higher perceived COVID-19 threat. Cronbach’s *α* was 0.72.

### Data Analysis

Prior to conducting the analysis, data normality was assessed. No continuous variables exhibited non-normal distribution (skewness values > |2|, [52]). We took three steps to examine proposed hypotheses. First, bivariate analysis (correlation for continuous predictors, independent sample t-test for binary predictor) was conducted to identify variables that had significant associations with psychiatric symptoms (i.e., anxiety, depression, and traumatic stress). Second, variables identified in the first step as having significant bivariate associations with psychiatric symptoms were entered to a regression model simultaneously in order to identify significant factors on a multivariate level (Model 1). A second model for each outcome only included variables significant *(p* < *.*05) in Model 1 (Model 2). This approach allows us to identify significant predictors, build a model with these factors (Model 2), and subsequently compare it with the regression model with the interaction term and controlled for significant covariates [2, 16]. Third, to investigate the interactive effects of perceived COVID-19 threat and societal COVID-19 stigma, we conducted multiple regression analyses examining their interaction covarying significant predictors from Model 2 (Model 3). All continuous predictors were mean-centered in all models to avoid potential multicollinearity and for ease of understanding (i.e., a one-unit difference means one SD difference). Gender as a binary variable was dummy coded (male = 0, female = 1). For models that yielded significant interaction effects, simple slopes analysis was employed to probe interactions at ±1 *SD* from the mean of the moderator [2]. Model comparison using ANOVA was conducted to further identify the proportion of variance explained by the interaction term. All regression analyses complied with assumptions regarding variable distribution and there was no evidence for collinearity (VIF values ranged from 1.02 to 1.50).

## Results

### Descriptive and bivariate statistics

The sample included 1912 Chinese university students. Average age was 20.28 (*SD* = 2.10, Median = 20, Range = [18, 49]). The majority of participants were female (*n* = 1334; 69.77%). Most participants (91.84%) were pursuing their undergraduate education. Participants resided in 30 out of the 36 provinces in China. Most participants noted some level of financial stress on their family due to COVID-19 (83%; Table 1). Students’ areas of study varied: 36.4% majored in medicine, 16.2% in science, 13.3% in engineering, 12.2% in economics, 8.5% in industrial organization, 6.8% in literature, 2.2% in art, 1.4% in education, 1.3% in law, 1.1% in history, and 0.4% in agriculture.

**Table 1:** Sample characteristics

Sociodemographic variables	***N*** (%)
**Gender**	
Female	1334 (69.77)
Male	578 (30.23)
**Socioeconomic status compared to classmates in school**	
Lower than average	675 (35.30)
On average	1166 (60.98)
Higher than average	71 (3.71)
**Financial stress on family due to COVID-19**	
Very significant stress	70 (3.66)
Significant stress	300 (15.69)
Moderate stress	782 (40.90)
Little stress	435 (22.75)
No stress	325 (17.00)
**Education (currently pursuing)**	
Undergraduate	1756 (91.84)
Master’s education	139 (7.27)
Doctorate education	17 (0.89)
**Anxiety**	
No symptoms (0–4)	1248 (65.27)
Mild symptoms (5–9)	480 (25.10)
Moderate symptoms (10–14)	158 (8.26)
Severe symptoms (≥ 15)	26 (1.36)
**Depression**	
No symptoms (0–4)	1022 (53.45)
Mild symptoms (5–9)	592 (30.96)
Moderate symptoms (10–14)	169 (8.84)
Moderately severe (15–19)	102 (5.33)
Severe symptoms (20–27)	27 (1.41)
**Traumatic stress**	
No symptoms (0–8)	630 (32.95)
Mild symptoms (9–25)	944 (49.37)
Moderate symptoms (26–43)	119 (6.22)
Severe symptoms (≥ 44)	219 (11.45)
**Suicide ideation**	
Never	1538 (80.44)
Rarely	261 (13.65)
Sometimes	93 (4.86)
Often	20 (1.05)
**Region**	
North China (Beijing, Tianjin, Hebei, Shanxi, Inner Mongolia)	275 (14.38)
Northeast China (Liaoning, Jilin, Heilongjiang)	61 (3.19)
East China (Shanghai, Jiangsu, Zhejiang, Anhui, Fujian, Jiangxi, Shandong)	541 (28.29)
South Central China (Henan, Hubei, Hunan, Guangdong, Guangxi, Hainan)	856 (44.77)
Southwest China (Chongqing, Sichuan, Guizhou, Yunan, Tibet)	109 (5.70)
Northwest China (Shaanxi, Gansu, Qinghai, Ningxia, Xinjiang)	67 (3.50)
Hong Kong, Macau, or living abroad	3 (0.16)

Psychiatric symptoms were notably prevalent. The majority of participants (67.05%) reported COVID-19-related traumatic stress symptoms within the clinical range (i.e., mild or higher). Anxiety symptoms were clinical elevated among 34.73% participants and 46.55% for depression. Approximately one in five (19.56%) reported some degree of suicidal ideation in the past 2 weeks (from rarely to often). Most clinical elevations were in the mild range. Proportion with moderate or higher clinical elevations were 17.67% for traumatic stress, 15.58% for depression, and 9.62% for anxiety.

Socioeconomic status, family financial stress due to COVID-19, mindfulness, perceived social support, COVID-19 self-efficacy, perceived COVID-19 societal stigma, and perceived COVID-19 threat had significant associations with all three outcomes (Table 2). As socioeconomic status and family financial stress due to COVID-19 were correlated (*r* = − .43, *p* < .001) and measured related constructs, only family financial stress due to COVID-19 was used as a predictor in regression. Age was weakly positively associated with anxiety. COVID-19 prosocial behavior and screen media usage were significantly associated with anxiety and depression. Anxiety symptoms were higher for female participants (*t* [1017] = − 2.10, *p* = .036, mean [*M*] for male = 3.27, *M* for female = 3.74). Depressive symptoms and traumatic stress did not differ based on gender. Symptom severity across psychiatric domains did not differ based on region of residence (Hubei, the hotspot of COVID-19 or non-Hubei).

**Table 2:** Correlations among continuous variables

	Depression	Anxiety	Traumatic stress	Age	SES	Financial stress due to COVID-19	Mindfulness	Social support	COVID-19 prosocial behavior	COVID-19 self-efficacy	Perceived COVID-19 stigma	Perceived COVID-19 threat	Screen media usage
Anxiety	.69***	–											
Traumatic stress	.47***	.43***	–										
Age	.04	.06**	.03	–									
SES	−.15***	−.13***	−.09***	−.07**	–								
Financial stress due to COVID-19	.24***	.24***	.20***	.08***	−.43***	–							
Mindfulness	−.39***	−.31***	−.29***	.02	.12***	−.20***	–						
Social support	−.30***	−.26***	−.11***	.01	.16***	−.17***	.28***	–					
COVID-19 Prosocial behavior	−.13***	−.10***	.01	.03	.10***	−.06*	.21***	.47***	–				
COVID-19 efficacy	−.18***	−.20***	−.10***	.01	.12***	−.14***	.20***	.43***	.36***	–			
Perceived COVID-19 stigma	.37***	.32***	.42***	−.01	−.08***	.17***	−.24***	−.22***	−.14***	−.23***	–		
Perceived COVID-19 threat	.18***	.22***	.20***	−.08***	−.04	.09***	−.008***	−.01	.06***	.02	.14***	–	
Screen media usage	.11***	.06*	.04	−.10***	−.04	.03	−.10***	−.09***	−.07**	−.02	.02	.01	–
*M*	5.20	3.59	19.83	20.38	1.68	2.66	67.14	32.40	26.70	16.44	11.94	17.68	6.18
*SD*	5.33	4.35	15.98	2.10	0.54	1.05	13.27	5.86	5.61	2.50	3.98	3.52	3.05

*Note*. *p* < .05 *, *p* < .01 **, *p* < .001***

### Predicting anxiety symptoms

The final model without interaction term (Model 2) included three demographic variables (age: *β* = 0.073, gender: *β* = 0.045, financial stress due to COVID-19: *β* = 0.124), two known protective factors for anxiety symptoms including mindfulness (*β* = − 0.190) and social support (*β* = − 0.143), and four COVID-19 specific factors including COVID-19 prosocial behavior (*β* = 0.049), COVID-19 self-efficacy (*β* = − 0.055), perceived COVID-19 threat (*β* = 0.160), and perceived COVID-19 societal stigma (*β* = 0.199). Contrary to hypothesis, higher level of COVID-19 prosocial behavior positively associated with more anxiety symptoms.

**Fig. 1:**
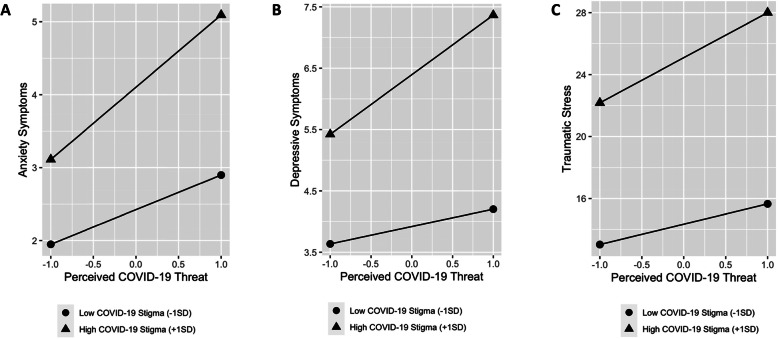
Interaction of Perceived COVID-19 threat X perceived COVID-19 societal stigma when predicting psychiatric symptoms

Accounting for all significant variables identified in Model 2, the interaction term of perceived COVID-19 threat X perceived COVID-19 societal stigma was significant in predicting anxiety symptoms (Table 3; Model 3), *β* = 0.070, *p* < .001. Main effects of perceived COVID-19 threat and perceived COVID-19 societal stigma remained significant. Model 3 was superior to model 2, *F* = 12.09, *p* < .001, accounting for 5% more variance. Simple slope analysis found that high levels of perceived COVID-19 societal stigma intensified the positive association between perceived COVID-19 threat and anxiety symptoms (*β* = 0.228 [0.173, 0.283], *p* < .001; Fig. 1a). At 1 *SD* below the mean of perceived COVID-19 societal stigma, it still heightened the detrimental effect of perceived COVID-19 threat on anxiety symptoms, *β* = 0.109 [0.060, 0.159], *p* < .001.

**Table 3:** Predictors of anxiety symptoms

	*B*	95%*CI*	*SE*	*beta*	*p*	Model *R*^2^	*F*
**Model 1**						.236	59.9
Age	0.327	[0.154, 0.501]	0.089	0.075	< .001***		
Gender	0.416	[0.039, 0.792]	0.192	0.044	.030*		
Financial stress due to COVID-19	0.538	[0.360, 0.716]	0.091	0.124	< .001***		
Mindfulness	−0.818	[−1.003, − 0.634]	0.094	− 0.188	< .001***		
Social support	− 0.614	[− 0.827, − 0.404]	0.107	− 0.141	< .001***		
COVID-19 prosocial behavior	0.216	[0.018, 0.415]	0.101	0.050	.033*		
COVID-19 self-efficacy	−0.244	[− 0.440, − 0.048]	0.100	− 0.056	< .001***		
Perceived COVID-19 threat	0.699	[0.524, 0.873]	0.089	0.161	< .001***		
Perceived COVID-19 societal stigma	0.867	[0.684, 1.049]	0.093	0.199	< .001***		
Screen media usage	0.109	[−0.064, 0.282]	0.088	0.025	.218		
**Model 2 (with significant variables identified in Model 1)**						.235	66.4
Age	0.317	[0.144, 0.490]	0.088	0.073	< .001***		
Gender	0.423	[0.047, 0.800]	0.192	0.045	.027*		
Financial stress due to COVID-19	0.539	[0.362, 0.717]	0.091	0.124	< .001***		
Mindfulness	−0.827	[−1.011, −0.643]	0.094	−0.190	< .001***		
Social support	−0.621	[−0.830, − 0.412]	0.106	−0.143	< .001***		
COVID-19 prosocial behavior	0.213	[0.014, 0.412]	0.101	0.049	.0236*		
COVID-19 self-efficacy	−0.240	[−0.436, − 0.044]	0.100	− 0.055	.016*		
Perceived COVID-19 threat	0.698	[0.523, 0.873]	0.089	0.160	< .001***		
Perceived COVID-19 societal stigma	0.866	[0.683, 1.048]	0.093	0.199	< .001***		
**Model 3 (with interaction)**						.240	61.3
Age	0.325	[0.152, 0.497]	0.088	0.075	< .001***		
Gender	0.424	[0.049, 0.799]	0.191	0.045	.027*		
Financial stress due to COVID-19	0.532	[0.355, 0.710]	0.090	0.122	< .001***		
Mindfulness	−0.839	[−1.022, −0.655]	0.094	−0.193	< .001***		
Social support	−0.623	[− 0.832, − 0.415]	0.106	−0.143	< .001***		
COVID-19 prosocial behavior	0.196	[−0.002, 0.394]	0.101	0.045	.053		
COVID-19 self-efficacy	−0.238	[− 0.433, − 0.043]	0.099	−0.055	.017*		
Perceived COVID-19 threat	0.733	[0.558, 0.909]	0.089	0.169	< .001***		
Perceived COVID-19 societal stigma	0.840	[0.658, 1.022]	0.093	0.193	< .001***		
COVID-19 threat X COVID-19 stigma	0.258	[0.112, 0.403]	0.074	0.070	< .001***		

*Note*. All continuous variables were mean-centered. *p* < .05 *, *p* < .01 **, *p* < .001***

### Predicting depressive symptoms

The final model without interaction (Model 2; Table 4) included six significant predictors of depressive symptoms. Risk factors (i.e., positive correlates) for depressive symptoms included financial stress due to COVID-19 (*β* = 0.119), two COVID-specific risk factors (perceived COVID-19 threat: *β* = 0.109; perceived COVID-19 societal stigma: *β* = 0.238), and screen media device usage (*β* = 0.058). Protective factors (i.e., negative correlates) for depressive symptoms included mindfulness (*β* = − 0.249) and perceived social support (*β* = − 0.153). The full model explained 28.2% of variance in depression (i.e., adjusted *R*^2^ = .282).

**Table 4:** Predictors of depressive symptoms

	*B*	95%*CI*	*SE*	*beta*	*p*	Model *R*^2^	*F*
**Model 1**						.286	95.2
Financial stress due to COVID-19	0.624	[0.413, 0.834]	0.107	0.117	< .001***		
Mindfulness	−1.348	[−1.567, −1.129]	0.112	−0.253	< .001***		
Social support	−0.898	[−1.146, − 0.650]	0.126	−0.168	< .001***		
COVID-19 prosocial behavior	0.212	[−0.024, 0.447]	0.120	0.040	.078		
COVID-19 self-efficacy	−0.030	[− 0.263, 0.202]	0.118	− 0.006	.799		
Perceived COVID-19 threat	0.570	[0.363, 0.776]	0.105	0.107	< .001***		
Perceived COVID-19 societal stigma	1.274	[1.058, 1.491]	0.110	0.239	< .001***		
Screen media usage	0.314	[0.109, 0.518]	0.104	0.059	.003**		
**Model 2 (with significant variables identified in Model 1)**						.282	126.4
Financial stress due to COVID-19	0.632	[0.422, 0.842]	0.107	0.119	< .001***		
Mindfulness	−1.329	[−1.547, −1.111]	0.111	−0.249	< .001***		
Social support	−0.817	[−1.033, − 0.602]	0.110	− 0.153	< .001***		
Perceived COVID-19 threat	0.583	[0.378, 0.789]	0.105	0.109	< .001***		
Perceived COVID-19 societal stigma	1.272	[1.057, 1.486]	0.109	0.238	< .001***		
Screen media usage	0.307	[0.103, 0.511]	0.104	0.058	.003**		
**Model 3 (with interaction)**						.288	111.3
Financial stress due to COVID-19	0.622	[0.413, 0.832]	0.107	0.117	< .001***		
Mindfulness	−1.347	[−1.564, −1.130]	0.111	−0.252	< .001***		
Social support	−0.829	[−1.044, − 0.614]	0.110	− 0.155	< .001***		
Screen media usage	0.311	[0.108, 0.515]	0.104	0.058	.003**		
Perceived COVID-19 threat	0.627	[0.421, 0.833]	0.105	0.118	< .001***		
Perceived COVID-19 societal stigma	1.238	[1.023, 1.452]	0.109	0.233	< .001***		
COVID-19 threat X COVID-19 stigma	0.344	[0.172, 0.517]	0.088	0.076	< .001***		

*Note*. All continuous variables were mean-centered. *p* < .05 *, *p* < .01 **, *p* < .001***

Perceived COVID-19 threat and perceived COVID-19 societal stigma had significant interaction (Model 3, *β* = 0.076, *p* < .001), after accounting for all other significant variables identified in Model 2. As predicted, the positive association between perceived COVID-19 threat with depressive symptoms was amplified in the presence of higher levels of perceived COVID-19 societal stigma. Model comparison indicated superiority of Model 3 to Model 2, *F* = 15.38, *p* < .001, and the interaction term accounted for 6% additional variance. Simple slope analyses indicated that perceived COVID-19 threat remained a significant predictor of depression when perceived COVID-19 societal stigma was 1 *SD* above (*β* = 0.182 [0.129, 0.235], *p* < .001) or below the mean (*β* = 0.053 [0.005, 0.101], *p* = .029), although depression was notably higher when perceived COVID-19 societal stigma was increased (Fig. 1b).

### Predicting traumatic stress

COVID-19 related traumatic stress was significantly predicted by financial stress due to COVID-19 (*β* = 0.100), mindfulness (*β* = − 0.178), perceived COVID-19 threat (*β* = 0.136), and perceived COVID-19 societal stigma (*β* = 0.341) (Table 5; Model 2). Model’s adjusted *R*^2^ = 0.238, *F* = 150.0, *p* < .001 (Table 5; Model 2).

**Table 5:** Predictors of traumatic stress

	*B*	95%*CI*	*SE*	*beta*	*p*	Model *R*^2^	*F*
**Model 1**						.238	100.7
Financial stress due to COVID-19	1.675	[1.026, 2.323]	0.331	0.105	< .001***		
Mindfulness	−2.995	[−3.666, −2.324]	0.342	−0.187	< .001***		
Social support	0.602	[−0.114, 1.318]	0.365	0.038	.100		
COVID-19 self-efficacy	0.123	[−0.581, 0.827]	0.356	0.008	.732		
Perceived COVID-19 threat	1.976	[1.341, 2.612]	0.324	0.124	< .001***		
Perceived COVID-19 societal stigma	5.570	[4.902, 6.238]	0.341	0.349	< .001***		
**Model 2 (with significant variables identified in Model 1)**						.238	150.0
Financial stress due to COVID-19	1.604	[0.959, 2.249]	0.329	0.100	< .001***		
Mindfulness	−2.844	[−3.498, − 2.190]	0.333	−0.178	< .001***		
Perceived COVID-19 threat	2.011	[1.377, 2.645]	0.323	0.136	< .001***		
Perceived COVID-19 societal stigma	5.450	[4.797, 6.104]	0.333	0.341	< .001***		
**Model 3 (with interaction)**						.241	122.2
Financial stress due to COVID-19	1.584	[0.941, 2.228]	0.328	0.099	< .001***		
Mindfulness	−2.892	[−3.546, −2.239]	0.333	−0.181	< .001***		
Perceived COVID-19 threat	2.113	[1.476, 2.749]	0.324	0.132	< .001***		
Perceived COVID-19 societal stigma	5.375	[4.721, 6.029]	0.335	0.336	< .001***		
COVID-19 threat X COVID-19 stigma	0.801	[0.268, 1.333]	0.271	0.059	.003**		

*Note*. All continuous variables were mean-centered. *p* < .05 *, *p* < .01 **, *p* < .001***

Accounting for significant variables identified in Model 2, as well as main effects of perceived COVID-19 threat and perceived COVID-19 societal stigma, their interaction term significantly predicted traumatic stress, *β* = 0.059. The model accounted for 24.1% of the variance in traumatic stress, *F* = 122.2, *p* < .001. Model 3 was superior to model 2, *F* = 8.70, *p* = .003, and accounted for 3% additional variance. Simple slope analysis revealed similar patterns: high levels of perceived COVID-19 societal stigma intensified the positive association between perceived COVID-19 threat and traumatic stress (*β* = 0.182 [0.128, 0.237], *p* < .001; Fig. 1c). At 1 *SD* below the mean of perceived COVID-19 societal stigma, it still heightened the positive association between perceived COVID-19 threat and traumatic stress, though to a less degree (*β* = 0.082 [0.033, 0.131], *p* = .001).

## Discussion

Results of this large-scale, cross-sectional study are largely consistent with prior studies in China and elsewhere highlighting the adverse psychological consequences in the general population of both quarantine and disease outbreak [10, 14, 57]. This study extends prior work by highlighting several risk and protective factors related to mental health in the context of the COVID-19 pandemic. Across symptoms domains (anxiety, depression, traumatic stress), COVID-related financial stress, perceived threat, and perceived societal stigma emerged as consistent positive correlates of psychopathology. Perceived COVID-19 threat and COVID-19 societal stigma showed synergistic interaction, such that the presence of both were associated with additive effects when predicting all three symptom domains, highlighting the importance of contextual factors (i.e., ecological perspective [9];). Both mindfulness and perceived social support emerged as protective factors, with mindfulness negatively associated with all three forms of psychopathology and perceived social support negatively associated with anxiety and depression. Screen media usage was weakly associated with depressive symptoms.

The prevalence on anxiety, depression, and traumatic stress among university students uncovered from this study is similar yet slightly higher than findings reported in two other recent studies concerning the initial phase of the outbreak [14, 57]. The prevalence of clinically elevated depressive symptoms in our sample (46.6%) is roughly twice as high as meta-analytic estimates of clinical elevations among Chinese college students prior to the pandemic (23.8%) [36]. To our knowledge, no meta-analyses or nationally representative survey data are available estimating rates of clinically elevated anxiety or traumatic stress symptoms among Chinese university students. Prevalence rates indicate continued mental health needs within this population during the early phase of the pandemic.

The COVID-19 pandemic has evolved into a serious threat to public health given its geographic reach, prolonged impact, and lack of cure or effective treatment. As hypothesized, perceived COVID-19 threat, namely the felt sense of threat to one’s health and life by COVID-19, predicted elevated psychiatric symptoms, highlighting the role of health concerns in psychological health during the pandemic. Public health interventions may need to balance strategies to increase preventive behaviors against COVID-19 (e.g., social distancing, wearing masks) while attending to potentially detrimental mental health effects due to an increased sense of COVID-19 threat.

The significant role of perceived societal stigma on mental health, both alone and in interaction with perceived threat, is a novel and potentially important finding. Research on infectious disease stigma has focused on those infected or most vulnerable to infection (e.g., anticipated HIV stigma among gay and bisexual men) [55]. However, our study found that in the context of COVID-19, perceived societal stigma adversely affects the mental health of the general public (i.e., university students, not a group particularly vulnerable for infection or death). The mechanism through which perceived COVID-19 societal stigma operates to effect mental health is unclear; it might be related to the potential deteriorating impact of societal stigma on people’s collective self-esteem and societal belonging, as well as increased survivor guilt and empathic concerns for those affected in a high stigma environment [45, 58]. Further, perceived COVID-19 stigma and perceived COVID-19 threat had a synergetic effect on all mental health outcomes, supporting an ecological perspective (person X context) [9, 17] in understanding and addressing consequences of stigma.

As the economic consequences of COVID-19 have continued to unfold, it has become clear that individuals from lower socioeconomic background have been disproportionately burdened across the globe [1, 12]. In keeping, we found that family financial stress due to COVID-19 consistently predicted psychiatric symptoms. This relationship is not new; past economic recessions have witnessed increased rates of common mental disorders and suicidal behavior in other global regions [44, 50]. In addition to financial stress, being older and female were also identified as risk factors of anxiety symptoms. Students who are older may experience more pandemic-related stress related to employment and career development. Female students reported greater levels of anxiety, which is consistent with existing evidence that women report greater anxiety and more likely to develop anxiety disorders than men [40].

Mindfulness was identified as a protective factor across all three symptoms domains. In the midst of great uncertainty, anxiety, and despair during the COVID-19 pandemic, dispositional attentiveness to the present moment may protect young adults from excessive worries, rumination, and fear. This is supported by evidence suggesting decreased rumination may be one of the key mechanisms underlying the efficacy of mindfulness-based interventions [26]. Perceived social support also emerged as a protective factor for anxiety and depression. Loneliness and social isolation from peers may contribute to heightened distress for young adults during quarantine.

Excessive screen time has been linked to a variety of health concerns including obesity, sleep disturbance, and mental health issues [7, 22]. In our sample, participants’ self-reported an average of 6 h of daily screen media time outside of school and work purposes in the past 2 weeks. In the context of the pandemic, young adults may consume more screen media due to restricted access to other avenues of entertainment, increased media exposure related to COVID-19 (e.g., news, report), and the need for connection with peers. Screen media usage weakly predicted depression but not other symptoms. It is possible that screen media engagement may have a mixed role during the pandemic. For instance, videoconferencing with friends could be important to enhance social support and mental health while excessive TV watching by oneself may increase risk.

### Study limitations and strengths

The current study has several limitations. First, although the current study recruited a geographically diverse national sample compared to previous studies in this area of research [14, 57, 59], the open recruitment method via the internet has its drawbacks. In particular, those highly impacted by COVID-19 may be particularly interested in participation, which could upwardly bias estimates of psychiatric symptom severity. Similar to previous studies regarding mental health of university students during the pandemic [14, 57, 59], our sample was disproportionately female participants (69.77%). Potentially, female students may be more willing to voluntarily participate in these research projects. The higher proportion of women could have skewed the estimates in anxiety, given the higher prevalence of anxiety in female students. Additionally, the gender makeup and recruitment method may lead to findings not being representative of the larger Chinese university student population. Second, given the study’s cross-sectional method, causal directions of the observed relationships cannot be ascertained. For instance, COVID-19 prosocial behavior was associated with heightened anxiety, and this could be due to those who experience higher anxiety during the pandemic being more likely to engage in prosocial actions as a coping. Future longitudinal and experimental studies may further explore these associations. Third, the Impact of Events Scale (IES) [30] only measured two clusters of PTSD symptoms (intrusion and avoidance), missing negative alternations in cognition or mood and hyperarousal symptoms. Fourth, it would have been preferable to use clinician-rated measures to more accurately define prevalence rates. Similarly, given the self-report method, all variables are at risk to known biases (e.g., social desirability).

We also note several strengths. We collected a large, geographically diverse sample during the early phase of the COVID-19 pandemic and included a variety of psychiatric symptoms. Importantly, as current knowledge regarding psychological health during the COVID-19 pandemic is largely descriptive [14, 57], this research represents an initial effort to identify risk and protective factors, which is critical to our understanding and addressing of the psychological consequences of the COVID-19 pandemic. Informed by previous research on infectious disease (e.g., HIV) [6], we investigated the roles of psychosocial variables specific to COVID-19 to inform our knowledge on the mental health of a general population (i.e., university students) during massive lockdown in the midst of a rapidly spreading disease. Further, the significant interaction between perceived COVID-19 societal stigma and personal sense of COVID-19 threat informs a theory-driven, ecological perspective in understanding the role of stigma on psychiatric symptoms during an emerging pandemic.

### Implications for research and clinical interventions

This study highlights several directions for future research. First, longitudinal research is needed to examine the trajectory of psychiatric symptoms over time as the COVID-19 pandemic continues to evolve. Due to China’s success in containing the outbreak [51], most universities have resumed in-person classes in May 2020, yet caution regarding potential COVID-19 outbreaks and public health policies such as mandate on mask wearing remain [42]. It is possible that the unintended psychological consequences of lockdowns, as we documented in this paper, may be reduced in some individuals as concerns over the pandemic lessen whereas in others, leaving untreated, psychiatric symptoms may continue to persist. Longitudinal research is also ideal to investigate how risk and protective factors identified in this study may affect psychological health over time. Second, findings suggest new areas of research concerning stigma and health in the context of pandemic-related stress. As noted by Allport [3], stigma is derived from the separation of human groups (“us” vs. “them”) and stereotypes associated with group membership [3]. Thus far, stigma research has focused on victimized groups. Findings from this study highlight the relevance of stigma in the mental health of the general public during an infectious disease outbreak. It is possible that stigmatizing attitudes harm everyone, though the degree of impact may vary based on perceived infection likelihood to the individual (i.e., ecological perspective). It is also plausible that due to the highly transmissible nature and large-scale impact of COVID-19, the psychological experience and consequences of COVID-19 societal stigma among the general public is fundamentally different from stigma based on other types of group memberships that may be perceived as more fixated states (e.g., race, sexual orientation, HIV/AIDS). Future studies are needed to clarify these relationships.

Most mental health systems in LMIC context, including in China, are often overburdened by high demand yet inadequately funded [63]. While there have been efforts to address the mental health crisis during the pandemic in China and other global regions, they have often largely focused on psychiatric patients and frontline health workers [64]. Findings of the study highlight the need to promote mental health among university students during a large-scale quarantine and offer insights to public health policy and intervention strategies in the LMIC context. First, there is a high need address the highly prevalent psychiatric symptoms among university students during quarantine. National and local governments, public health officials, and educational units may work together to raise public awareness of mental health issues in young adults. Policy makers and stakeholders need to allocate resources to strengthen the mental health system for university students. University health services should consider outreach efforts to effectively reach those in need, such as via websites, webinars and internet-based psychoeducation programs, and mobile health-based interventions [38]. Given the increased burden of care for mental health professionals, especially in the LMIC context, stakeholders may consider efficient and empirically-proven approaches to address the high volume of mental health need. This may include training lay mental health providers and organization of peer support groups [53]. Second, mental health interventions targeting multi-level factors, including promoting mindfulness (e.g., via internet-based mindfulness programs) [37] and social support at individual and interpersonal levels while reducing societal stigma, may be particularly promising. Lessons from stigma reduction for other pandemics, such as HIV/AIDS [21], can inform such efforts in the context of well-being and disease prevention for COVID-19. For instance, public health professionals may use strategies proven to be effective in addressing stigma of other infectious diseases globally, such as anti-stigma campaigns, crowdsourcing, and community engagement. Third, attending to the needs of disadvantaged groups is important, including those who are financially vulnerable. Given the dual burden of financial stress and COVID-19 infection vulnerability, combination of both psychological and economic interventions may be warranted.

## Conclusions

Assessing psychiatric symptom prevalence and identifying risk and protective factors during the COVID-19 outbreak are critical to understanding and addressing the short-term and long-term psychological consequences of the COVID-19 pandemic. In addition to informing current and future pandemic responses, this research can also clarify the psychological consequences, risk, and protective factors of acute stress more generally. Conducted in March and April 2020 during massive, state-enforced quarantine, the current study found high rates of psychiatric symptoms in a sample of internet-recruited university students in China. Findings highlight the high need for psychological health promotion in this population. In addition, findings inform an ecological-driven understanding regarding the detrimental role of public stigma on psychological health in the general population during an emerging infectious disease outbreak. Interventions targeting multi-level factors, including promoting mindfulness and social support at individual and interpersonal levels while reducing societal stigma, may be particularly promising. Attending to needs of disadvantaged groups most financially vulnerable may require both psychological and economic interventions.

## Data Availability

All data associated with this paper will be made available upon request.

## References

[1] Ahmed F, Ahmed N, Pissarides C, Stiglitz J (2020). Why inequality could spread COVID-19. Lancet Public Health.

[2] Aiken LS, West SG (1991). Multiple regression: Testin and interpreting interactions.

[3] Allport GW (1954). The nature of prejudice.

[4] American Psychiatric Association (1994). Diagnostic and statistical manual of mental disorders: DSM-IV.

[5] Arnett JJ, Žukauskiene R, Sugimura K (2014). The new life stage of emerging adulthood at ages 18-29 years: implications for mental health. Lancet Psychiatry.

[6] Berger BE, Ferrans CE, Lashley FR (2001). Measuring stigma in people with HIV: psychometric assessment of the HIV stigma scale. Res Nurs Health.

[7] Boone JE, Gordon-Larsen P, Adair LS, Popkin BM (2007). Screen time and physical activity during adolescence: longitudinal effects on obesity in young adulthood. Int J Behav Nutr Phys Act.

[8] Bovier PA, Chamot E, Perneger TV (2004). Perceived stress, internal resources, and social support as determinants of mental health among young adults. Qual Life Res.

[9] Bronfenbrenner U (1977). Toward an experimental ecology of human development. Am Psychol.

[10] Brooks SK, Webster RK, Smith LE, Woodland L, Wessely S, Greenberg N, Rubin GJ (2020). The psychological impact of quarantine and how to reduce it: rapid review of the evidence. Lancet.

[11] Brown KW, Ryan RM (2003). The benefits of being present: mindfulness and its role in psychological well-being. J Pers Soc Psychol.

[12] Buheji M, da Costa Cunha K, Beka G, Mavrić B, Leandro do Carmo de Souza Y, Souza da Costa Silva S, Hanafi M, Chetia Yein T (2020). The extent of COVID-19 pandemic socio-economic impact on global poverty: a global integrative multidisciplinary review. Am J Econ.

[13] Cahyanto I, Wiblishauser M, Pennington-Gray L, Schroeder A (2016). The dynamics of travel avoidance: the case of Ebola in the U.S. Tour Manag Perspect.

[14] Cao W, Fang Z, Hou G, Han M, Xu X, Dong J, Zheng J (2020). The psychological impact of the COVID-19 epidemic on college students in China. Psychiatry Res.

[15] Caprara GV, Steca P, Zelli A, Capanna C (2005). A new scale for measuring adults’ prosocialness. Eur J Psychol Assess.

[16] Cohen J, Cohen P, West SG, Aiken LS (2003). Data-analytic strategies using multiple regression/correlation, in: applied multiple regression/correlation analysis for the behavioral sciences.

[17] Cook JE, Purdie-Vaughns V, Meyer IH, Busch JTA (2014). Intervening within and across levels: a multilevel approach to stigma and public health. Soc Sci Med.

[18] Curry OS, Rowland LA, Van Lissa CJ, Zlotowitz S, McAlaney J, Whitehouse H (2018). Happy to help? A systematic review and meta-analysis of the effects of performing acts of kindness on the well-being of the actor. J Exp Soc Psychol.

[19] Deng YQ, Li S, Tang YY, Zhu LH, Ryan R, Brown K (2012). Psychometric properties of the Chinese translation of the mindful attention awareness scale (MAAS). Mindfulness (N Y).

[20] Des Jarlais DC, Galea S, Tracy M, Tross S, Vlahov D (2006). Stigmatization of newly emerging infectious diseases: AIDS and SARS. Am. J. Public Health.

[21] Eaton LA, Kalichman SC (2020). Social and behavioral health responses to COVID-19: lessons learned from four decades of an HIV pandemic. J Behav Med.

[22] Feng Q, Le Zhang Q, Du Y, Ye YL, He QQ (2014). Associations of physical activity, screen time with depression, anxiety and sleep quality among Chinese college freshmen. PLoS One.

[23] Fiksenbaum L, Marjanovic Z, Greenglass ER, Coffey S (2006). Emotional exhaustion and state anger in nurses who worked during the SARS outbreak: the role of perceived threat and organizational support. Can J Community Ment Heal.

[24] Garfin DR, Silver RC, Holman EA (2020). The novel coronavirus (COVID-2019) outbreak: amplification of public health consequences by media exposure. Health Psychol.

[25] Goldberg SB, Tucker RP, Greene PA, Davidson RJ, Wampold BE, Kearney DJ, Simpson TL (2018). Mindfulness-based interventions for psychiatric disorders: a systematic review and meta-analysis. Clin Psychol Rev.

[26] Gu J, Strauss C, Bond R, Cavanagh K (2015). How do mindfulness-based cognitive therapy and mindfulness-based stress reduction improve mental health and wellbeing? A systematic review and meta-analysis of mediation studies. Clin Psychol Rev.

[27] He J, He L, Zhou W, Nie X, He M (2020). Discrimination and social exclusion in the outbreak of COVID-19. Int J Environ Res Public Health.

[28] Hefner J, Eisenberg D (2009). Social support and mental health among college students. Am J Orthop.

[29] Holmes EA, O’Connor RC, Perry VH, Tracey I, Wessely S, Arseneault L, Ballard C, Christensen H, Cohen Silver R, Everall I, Ford T, John A, Kabir T, King K, Madan I, Michie S, Przybylski AK, Shafran R, Sweeney A, Worthman CM, Yardley L, Cowan K, Cope C, Hotopf M, Bullmore E. Multidisciplinary research priorities for the COVID-19 pandemic: a call for action for mental health science. Lancet Psychiatry. 2020:547–60. 10.1016/S2215-0366(20)30168-1. 10.1016/S2215-0366(20)30168-1 PMC7159850 32304649

[30] Horowitz M, Wilner N, Alvarez W (1979). Impact of event scale: a measure of subjective stress. Psychosom Med.

[31] Kabat-Zinn J (1994). Wherever you go, there you are: mindfulness meditation in everyday life.

[32] Keng SL, Smoski MJ, Robins CJ (2011). Effects of mindfulness on psychological health: a review of empirical studies. Clin Psychol Rev.

[33] Kroenke K, Spitzer RL, Williams JBW (2001). The PHQ-9: validity of a brief depression severity measure. J Gen Intern Med.

[34] Kurzban R, Burton-Chellew MN, West SA (2015). The evolution of altruism in humans. Annu Rev Psychol.

[35] Lee-Baggley D, DeLongis A, Voorhoeave P, Greenglass E (2004). Coping with the threat of severe acute respiratory syndrome: role of threat appraisals and coping responses in health behaviors. Asian J Soc Psychol.

[36] Lei XY, Xiao LM, Liu YN, Li YM (2016). Prevalence of depression among Chinese university students: a neta-analysis. PLoS One.

[37] Linardon J (2020). Can acceptance, mindfulness, and self-compassion be learned by smartphone apps? A systematic and meta-analytic review of randomized controlled trials. Behav Ther.

[38] Linardon J, Cuijpers P, Carlbring P, Messer M, Fuller-Tyszkiewicz M (2019). The efficacy of app-supported smartphone interventions for mental health problems: a meta-analysis of randomized controlled trials. World Psychiatry.

[39] Martela F, Ryan RM (2016). Prosocial behavior increases well-being and vitality even without contact with the beneficiary: causal and behavioral evidence. Motiv Emot.

[40] McLean CP, Anderson ER (2009). Brave men and timid women? A review of the gender differences in fear and anxiety. Clin Psychol Rev.

[41] Ministry of Education of China, 2020a. Notification on delayed school opening in spring semester 2020 [WWW document]. Minist. Educ. China. URL http://www.moe.gov.cn/srcsite/A06/s3321/202002/t20200212_420435.html (Accessed 11.11.20).

[42] Ministry of Education of China, 2020b. Guidelines on re-opening educational system and COVID-19 prevention in schools [WWW document]. Minist. Educ. China. URL http://www.moe.gov.cn/fbh/live/2020/51974/sfcl/202005/t20200512_452977.html (Accessed 11.11.20).

[43] Ministry of Education of China and Ministry of Industry and Information Technology of China, 2020. Notice of Arrangement for “Suspension of School and Continued Learning” During the Postponement for The Opening of Primary and Secondary Schools [WWW Document].

[44] Nordt C, Warnke I, Seifritz E, Kawohl W (2015). Modelling suicide and unemployment: a longitudinal analysis covering 63 countries, 2000-11. Lancet Psychiatry.

[45] O’Connor LE, Berry JW, Weiss J, Gilbert P (2002). Guilt, fear, submission, and empathy in depression. J Affect Disord.

[46] Overholt L, Wohl DA, Fischer WA, Westreich D, Tozay S, Reeves E, Pewu K, Adjasso D, Hoover D, Merenbloom C, Johnson H, Williams G, Conneh T, Diggs J, Buller A, McMillian D, Hawks D, Dube K, Brown J (2018). Stigma and Ebola survivorship in Liberia: results from a longitudinal cohort study. PLoS One.

[47] Patel V, Flisher AJ, Hetrick S, McGorry P (2007). Mental health of young people: a global public-health challenge. Lancet.

[48] Person B, Sy F, Holton K, Govert B, Liang A, the NCID/SARS Community Outreach Team (2004). Fear and stigma: the epidemic within the SARS outbreak. Emerg Infect Dis.

[49] Pfefferbaum B, North CS. Mental health and the COVID-19 pandemic. N Engl J Med. 2020. 10.1056/NEJMp2009027. 10.1056/NEJMp2008017 32283003

[50] Reeves A, Stuckler D, McKee M, Gunnell D, Chang SS, Basu S (2012). Increase in state suicide rates in the USA during economic recession. Lancet.

[51] Salzberger B, Glück T, Ehrenstein B (2020). Successful containment of COVID-19: the WHO-report on the COVID-19 outbreak in China. Infection.

[52] Schminder E, Ziegler M, Danay E, Beyer L, Bühner M (2010). Is it really robust? Reinvestigating the robustness of ANOVA against violations of the normal distribution. Eur Res J Methods Behav Soc Sci.

[53] Singla DR, Kohrt BA, Murray LK, Anand A, Chorpita BF, Patel V (2017). Psychological treatment for the world: lessons from low- and middle-income countries. Annu Rev Clin Psychol.

[54] Spitzer RL, Kroenke K, Williams JB, Lowe B (2006). A brief measure for assessing generalized anxiety disorder: the GAD-7. Arch Intern Med.

[55] Stangl AL, Lloyd JK, Brady LM, Holland CE, Baral S (2013). A systematic review of interventions to reduce HIV-related stigma and discrimination from 2002 to 2013: how far have we come?. J Int AIDS Soc.

[56] Sun, S., Lin, D., Operario, D., 2020. Need for a population health approach to understand and address psychosocial consequences of COVID-19. Psychol Trauma Theory Res Pract Policy. doi: 10.1037/tra0000618. 10.1037/tra0000618 PMC8300874 32496107

[57] Tang W, Hu T, Hu B, Jin C, Wang G, Xie C, Chen S, Xu J (2020). Prevalence and correlates of PTSD and depressive symptoms one month after the outbreak of the COVID-19 epidemic in a sample of home-quarantined Chinese university students. J Affect Disord.

[58] Taylor S (2019). The psychology of pandemics: preparing for the next global outbreak of infectious disease.

[59] Wang C, Zhao H (2020). The impact of COVID-19 on anxiety in Chinese University students. Front Psychol.

[60] Wang L, Zhang J, Shi Z, Zhou M, Huang D, Liu P (2011). Confirmatory factor analysis of posttraumatic stress symptoms assessed by the impact of event scale-revised in Chinese earthquake victims: examining factor structure and its stability across sex. J Anxiety Disord.

[61] Wang W, Bian Q, Zhao Y, Li X, Wang W, Du J, Zhang G, Zhou Q, Zhao M (2014). Reliability and validity of the Chinese version of the patient health questionnaire (PHQ-9) in the general population. Gen Hosp Psychiatry.

[62] World Health Organization. Mental health and psychosocial considerations during COVID-19 outbreak: World Health Organization; 2020. https://www.who.int/docs/default-source/coronaviruse/mental-health-considerations.pdf.

[63] World Health Organization. Mental health atlas 2017: World Health Organization; 2017. https://apps.who.int/iris/bitstream/handle/10665/272735/9789241514019-eng.pdf?ua=1.

[64] Xiang Y, Jin Y, Cheung T (2020). Joint international collaboration to combat mental health challenges during the coronavirus disease 2019 pandemic. JAMA Psychiatry.

[65] Zeng Q-Z, He Y-L, Liu H, Miao J-M, Chen J-X, Xu H-N, Wang J-Y (2013). Reliability and validity of Chinese version of the generalized anxiety disorder 7-item (GAD-7) scale in screening anxiety disorders in outpatients from traditional Chinese internal department. Chinese Ment Heal J.

[66] Zhao C, Wang X, Chang L (2003). Reliability and validity of the impact of event scale in Chinese traumatic event suffers. Chinese Ment Heal J.

[67] Zimet GD, Dahlem NW, Zimet SG, Farley GK (1988). The multidimensional scale of perceived social support. J Pers Assess.

