# Correction to: Psychiatric symptoms, risk, and protective factors among university students in quarantine during the COVID-19 pandemic in China

**DOI:** 10.1186/s12992-021-00701-8

**Published:** 2021-04-26

**Authors:** Shufang Sun, Simon B. Goldberg, Danhua Lin, Shan Qiao, Don Operario

**Affiliations:** 1 Department of Behavioral and Social Sciences, Brown University School of Public Health, Providence, RI USA; 2 Mindfulness Center at Brown University, Providence, RI USA; 3 Department of Counseling Psychology, University of Wisconsin-Madison College of Education, Madison, WI USA; 4 Institute of Developmental Psychology, Beijing Normal University, Beijing, China; 5 Department of Health Promotion, Education, and Behavior, University of South Carolina Arnold School of Public Health, Columbia, SC USA

**Correction to: Global Health 17, 15 (2021)**

**https://doi.org/10.1186/s12992-021-00663-x**

Following publication of the original article [1], it was brought to our attention that the article had been provided with incorrect affiliation details.

The affiliation details have been updated in the original article and the correct details can be seen in this correction.

The authors apologize for any inconvenience caused.

## References

[1] Sun S, Goldberg SB, Lin D, Qiao S, Operario D (2021). Psychiatric symptoms, risk, and protective factors among university students in quarantine during the COVID-19 pandemic in China. Glob Health.

